# The relationship between body appreciation and weight-related health behaviors among Chinese adolescents

**DOI:** 10.3389/fpsyg.2025.1484077

**Published:** 2025-01-29

**Authors:** Yuyuan Zhong, Ying Chen, Meng Wang, Bian Lu, Ting Shen, Jing Hu, Fengyang Jiang, Hongmei Wang

**Affiliations:** ^1^Department of Social Medicine of School of Public Health and Department of Pharmacy of the First Affiliated Hospital, Zhejiang University School of Medicine, Hangzhou, Zhejiang, China; ^2^Department of Public Health Emergency Response, Zhejiang Center for Disease Control and Prevention, Hangzhou, Zhejiang, China; ^3^Hangzhou Center for Disease Control and Prevention, Hangzhou, Zhejiang, China; ^4^Xiaoshan District Center for Disease Control and Prevention, Hangzhou, Zhejiang, China; ^5^Xihu District Center for Disease Control and Prevention, Hangzhou, Zhejiang, China

**Keywords:** body appreciation, weight-related health behaviors, physical activity, dietary behaviors, Chinese adolescents

## Abstract

**Introduction:**

Research on body appreciation has flourished globally in recent years. College students and adults, rather than adolescents have been mainly focused in China. Therefore, this study aims to investigate the current status of body appreciation and weight-related health behaviors among Chinese adolescents, and elucidate their multifaceted relationships.

**Methods:**

A total of 487 students from two junior high schools were selected through stratified cluster sampling for this cross-sectional study conducted in Hangzhou city, China. Data were collected by using the questionnaire including the Body Appreciation Scale-2 (BAS-2), the Physical Activity Questionnaire for Adolescents (PAQ-A), and the self-designed Food Frequency Questions.

**Results:**

The study showed current status of body appreciation among Chinese adolescents, with a majority categorized as physically inactive and having unhealthy dietary behaviors, leading to abnormal weight. Notably, this study provided novel evidence linking body appreciation with positive weight-related behavioral outcomes in both physical activity and dietary behaviors (such as sufficient fruit and vegetable consumption and reduced fried food intake) among Chinese adolescents. Besides, this study also revealed the impact of gender, mother's education, and household income on weight-related health behaviors among adolescents.

**Discussion:**

This study emphasized the significant relationships between body appreciation and weight-related health behaviors among Chinese adolescents. These findings present empirical evidence for the development of intervention programs aimed at fostering body appreciation and promoting weight-related health behaviors in adolescents.

## 1 Introduction

Body image is a complex and multidimensional construct that can affect quality of life, as well as cognitive, affective, and behavioral functioning (Cash et al., 1991), but it can be simply described as one's positive or negative feelings toward their own body shape (McClain and Peebles, 2016). Previously, research in the field focused on the negative aspects of body image, particularly body dissatisfaction (Carvalho et al., 2020; Gualdi-Russo et al., 2022; Jiménez Flores et al., 2017). However, recent evidence suggests that positive body image has a greater impact on various life domains than negative body image (Cash and Fleming, 2002). Body appreciation is the core component of positive body image, defined as accepting, respecting, and thinking favorably of the body despite its deviation from media-portrayed ideals (Tylka and Wood-Barcalow, 2015b).

Body appreciation has been proven to be positively associated with numerous health-promoting behaviors, including sports participation (Riddervold et al., 2023), lower eating pathology (Linardon et al., 2022) and adaptive eating patterns (Linardon, 2021). Additionally, body appreciation is inversely associated with health-compromising behaviors, including alcohol and tobacco consumption (Nolen and Panisch, 2022). Given this evidence, it is of vital significance for adolescents to maintain body appreciation. However, China Health and Nutrition Survey (CHNS) in 2015 (Song et al., 2022) has shown that nearly three-fifths of Chinese children and adolescents aged 6–17 years held a negative attitude toward their bodies. Therefore, the development of body appreciation should be attached great importance to assist adolescents in achieving optimal well-being.

Over the past few decades, there has been an increased prevalence of overweight and obesity among children and adolescents (Abarca-Gómez et al., 2017), with weight-related behaviors playing significant roles in either increasing or reducing the risk. Only 14.2% and 3.1% of Chinese children and adolescents aged 6–17 meet the minimum daily recommendation for fruit and vegetable intake (Li et al., 2020). The statistics for physical activity present an equally bleak picture. Only 3.7% of children and adolescents meet physical activity guidelines (engaging in moderate to vigorous physical activity for at least 1 h daily) (Wang et al., 2021). Based on this situation, weight-related health behaviors among Chinese children and adolescents deserve more attention.

In previous work, researchers observed associations between body appreciation and weight-related health behaviors (especially physical activity and dietary behaviors). Piko et al. (2020) revealed that among female adolescents, body appreciation was positively associated with self-perceived health, sports participation and controlled diets. Kantanista et al. (2015) revealed that body image had a statistically significant positive correlation with moderate to vigorous physical activity among teenagers. Similarly, a few studies in China also uncovered analogous outcomes, but the participants in these studies were primarily college students and adults, rather than adolescents. However, adolescence is a formative period for developing the body appreciation and weight-related behaviors that often persist into adulthood (Daly et al., 2022; Gattario and Frisén, 2019), making adolescence the optimal time to encourage body appreciation and healthier behaviors. Understanding the relationship between body appreciation and weight-related health behaviors during this period is crucial for developing targeted health strategies for adolescents. Existing domestic studies have mainly concentrated on the separate relationship between body appreciation and either physical activity or dietary behaviors, but there is limited research exploring the relationship between body appreciation and these critical weight-related health behaviors.

This study aims to investigate the current status of body appreciation and weight-related health behaviors (particularly physical activity and dietary behaviors) among adolescents, and further elucidate their multifaceted relationships.

## 2 Method

### 2.1 Participants and procedures

This cross-sectional study was conducted between September and October 2022 in Hangzhou, China. A multi-stage cluster sampling was used to recruit participants. Firstly, Xihu District and Xiaoshan District located in central and suburb of Hangzhou, respectively, were purposively selected as the study area. Secondly, one junior high school was selected randomly from each of the two districts. Within the grades 7th and 8th of the target schools, a cluster sampling was employed, three classes were randomly selected from each grade. Participants were excluded according to the following criteria: (1) suffered from morbid obesity, metabolic disorders, neuroendocrine diseases or congenital genetic disease; (2) refused to sign the informed consent, or was unable to complete the questionnaire.

After obtaining permission from school principals, our research team visited selected class to recruit sufficient participants. The research assistants introduced the study and explained the caveats of the questionnaire, and then supervised participants filling out the questionnaire independently after obtaining the written consent. A total of eligible 487 students participated in the study. After excluding invalid questionnaires with incomplete information or logical errors, we involved 464 valid respondents in the final data analysis, giving a response rate of 95.3%.

All study participants and their parents (or guardians) provided written informed consent at study entry. The study protocol was approved by the Ethics Committee of School of Public Health of Zhejiang University (No. ZGL202201-5).

### 2.2 Measures

Data were collected by a self-administrated questionnaire including demographic characteristics, body appreciation, physical activity, and dietary behaviors.

#### 2.2.1 Demographic characteristics

Participants self-reported their demographic information including gender, age, household registration, household income, and parents' education level. Weight and height were measured with an anthropometer, which were used to calculate body mass index (BMI) as kg/m^2^. BMI was categorized as underweight, normal weight, overweight, and obesity according to Screening standard for malnutrition of school-age children and adolescents (WS/T 456-2014) and the Screening for overweight and obesity among school-age children and adolescent (WS/T 586-2018) of China (Lei et al., 2019).

#### 2.2.2 Body appreciation

The Chinese version of the Body Appreciation Scale-2 (BAS-2) (Swami et al., 2016), originally developed by Tylka and Wood-Barcalow (2015a), was applied to assess body appreciation. This scale consisted of ten items and was rated on a 5-point Likert scale (1 = Never, 5 = Always). The average score of the scale was computed by taking the mean of all the items. Consequently, the range of scores varied from 1 to 5, with higher scores indicating greater body appreciation. Previous research showed that the scale had good reliability (Cronbach's alpha = 0.90) and validity in the Chinese sample (He et al., 2023). Similarly, the Cronbach's alpha for our present study stood at 0.92.

#### 2.2.3 Physical activity

We employed the Chinese version of the Physical Activity Questionnaire for Adolescents (PAQ-A) (Li et al., 2015) to assess the level of physical activity in the latest 7 days. The instrument comprised nine items. The first eight questions reflected the participation of physical activity at different times, such as during physical education class, at lunch, after school, during evenings and weekends, which were rated on a 5-point Likert scale, ranging from 1 (indicating low physical activity) to 5 (indicating high physical activity) (Wyszyńska et al., 2019). The ninth question informed us of the reasons preventing participants from engaging in physical activity during that week, as a basis for screening the valid questionnaires. The physical activity score was calculated from the average of the initial eight items. The higher the score, the greater the level of physical activity. Specifically, participants were categorized as active or inactive based on whether their scores were above or below 2.75, respectively (Benítez-Porres et al., 2016). This scale has been previously validated and employed in China (Li et al., 2019). In this study, the Cronbach's alpha was 0.89.

#### 2.2.4 Dietary behaviors

Dietary behaviors were measured by the simplified Food Frequency Questionnaire (FFQ) (Cheng et al., 2020) with culturally nuanced adaptations. All participants were required to report their average intake and consumption frequency for weight-related dietary categories (including fruit, vegetables, fried food, dessert, and sugary beverages) over the past month. These specific categories of food were chosen due to their prevalence in modern diets and their significant impact on health among adolescents. Fruit and vegetables are typically considered healthy, while fried food, dessert and sugary beverages are associated with unhealthy dietary patterns and obesity (Hong et al., 2023). The total intake of these foods was calculated by multiplying consumption frequency with average intake, subsequently converted to daily intake for further data analysis.

According to *Dietary Guidelines for Chinese Residents (2022)* (Chinese Nutrition Society, 2022), the recommended vegetable intake was 400–450 g/d for adolescents aged 11–17, and the recommended fruit intake was 200–300 g/d for adolescents aged 11–13, while 300–350 g/d for adolescents aged 14–17. In this study, sufficient fruit intake and sufficient vegetable intake were considered to meet the recommended standards of the Dietary Guidelines.

### 2.3 Statistical analysis

Descriptive statistics were performed to characterize the sample, concurrently testing for the normal distribution of continuous variables. Continuous data were presented as means and standard deviation (M ± SD) and median and interquartile [Median (IQR)], while categorical data were presented as frequencies (*N*) and percentages (%). The differences between groups were examined using the independent samples *t*-test or one-way ANOVA for parametric data, Mann-Whitney *U*-test or Kruskal-Wallis *H*-test for non-parametric data, and Pearson's chi-square test for categorical data.

The correlations between body appreciation, physical activity, and dietary behaviors were calculated using Pearson correlations or Spearman correlations. Hierarchical linear regression and logistic regression analyses were conducted to estimate the influence of body appreciation on physical activity and dietary behaviors, while controlling for covariates such as age, gender, household income, and parents' education level. Epidata 3.1 was used for data management and SPSS 26.0 for statistical analysis. Statistical significance was determined by a two-sided *p* < 0.05.

## 3 Results

### 3.1 Characteristic of participants

Participants' characteristics are presented in Table 1. In detail, 48.7% (*n* = 226) were boys, 48.9% (*n* = 227) were 14 years old and the majority (82.5%, *n* = 383) lived in rural areas. The education level of the father and mother was mainly middle or high school (76.1%, *n* = 353; 64.5%, *n* = 299, respectively). About 43.3% (*n* = 201) of participants reported that their average annual household income was 30,000–70,000 RMB. Approximately 70% of adolescents (*n* = 317) were of normal weight.

**Table 1:** Characteristics of participants (*n* = 464).

**Characteristics**	**Group**	***N* (%)**
Gender	Boys	226 (48.7)
	Girls	238 (51.3)
Age	Thirteen	143 (30.8)
	Fourteen	227 (48.9)
	Fifteen	92 (19.8)
Household registration	Urban	65 (14.0)
	Rural	383 (82.5)
Average annual household income (RMB)	< 30,000	59 (12.7)
	30,000–70,000	201 (43.3)
	>70,000	188 (40.5)
Father's education level	Primary school or below	27 (5.8)
	Middle School	204 (44.0)
	High school	149 (32.1)
	College or above	77 (16.6)
Mother's education level	Primary school or below	70 (15.1)
	Middle School	159 (34.3)
	High school	140 (30.2)
	College or above	89 (19.2)
Classification of BMI	Underweight	29 (6.3)
	Normal weight	317 (68.3)
	Overweight	62 (13.4)
	Obesity	54 (11.6)
Body appreciation, Mean ± SD		3.58 ± 0.88
Physical activity, Mean ± SD		1.96 ± 0.42
Classification of physical activity	Active	24 (5.2)
	Inactive	440 (94.8)
Fruit intake meets recommendation	Yes	121 (26.1)
	No	339 (73.1)
Vegetable intake meets recommendation	Yes	96 (20.7)
	No	363 (78.2)
Fried food intake, Median (IQR), g/d		6.7 (14.7)
Dessert intake, Median (IQR), g/d		10.7 (39.4)
Sugary beverage intake, Median (IQR), ml/d		36.9 (72.2)

*N*, number of participants; BMI, body mass index; SD, standard deviation; IQR, interquartile range.

In this study, the body appreciation and physical activity scores of the participants were 3.58±0.88 and 1.96±0.42, respectively. Only 5.2% (*n* = 24) of the adolescents were categorized as active, while 94.8% (*n* = 440) of them were categorized as inactive. In terms of dietary behaviors, a significant number of participants did not meet the recommended intake of fruit (73.1%, *n* = 339) and vegetables (78.2%, *n* = 363) according to the Dietary Guidelines (Chinese Nutrition Society, 2022). Besides, the Median (IQR) of fried food intake was 6.7 (14.7) g/d, dessert intake was 10.7 (39.4) g/d, and sugary beverage intake was 36.9 (72.2) ml/d.

### 3.2 Univariate analysis of physical activity and dietary behaviors

The normality test showed that the scores of body appreciation and physical activity were normally distributed, whereas the scores of the intake of fruit, vegetables, fried food, dessert, and sugary beverages were non-normal distribution.

As shown in Table 2, there were significant gender differences and parents' education level differences in physical activity among the surveyed respondents. Boys were more likely to take part in physical activity than girls (2.03 ± 0.42 and 1.89 ± 0.41, respectively, *p* < 0.001). In addition, the participants whose fathers had high school education had a higher level of physical activity than those whose fathers had middle school education (2.06 ± 0.45 and 1.93 ± 0.39, respectively, *p* =0.010). Similarly, the participants whose mothers had high school education had a higher level of physical activity than those whose mothers had primary school education or below (2.02 ± 0.43 and 1.86 ± 0.38, respectively, *p* =0.016).

**Table 2:** Univariate analysis of physical activity and dietary behaviors (*n* = 464).

**Characteristics**	**Group**	**Physical activity** ^**1**^		**Fruit intake meets recommendation** ^**2**^			**Vegetable intake meets recommendation** ^**2**^		
		**Mean** ±**SD**	***p***	**Yes**, ***N*** **(%)**	**No**, ***N*** **(%)**	***p***	**Yes**, ***N*** **(%)**	**No**, ***N*** **(%)**	***p***
Gender	Boys	2.03 ± 0.42	< 0.001^*^	58 (26.1)	164 (73.9)	0.933	50 (22.4%)	173 (77.6%)	0.440
	Girls	1.89 ± 0.41		63 (26.5)	175 (73.5)		46 (19.5%)	190 (80.5%)	
Age	Thirteen	2.01 ± 0.47	0.170	54 (38.0) ^a^	88 (62.0) ^a^	0.001^*^	29 (20.4%)	113 (79.6%)	0.932
	Fourteen	1.96 ± 0.38		50 (22.3)^b^	174 (77.7)^b^		46 (20.4%)	179 (79.6%)	
	Fifteen	1.91 ± 0.43		17 (18.5)^b^	75 (81.5)^b^		20 (22.2%)	70 (77.8%)	
Household registration	Urban	2.00 ± 0.48	0.529	19 (29.7)	45 (70.3)	0.513	11 (17.2%)	53 (82.8%)	0.445
	Rural	1.96 ± 0.41		98 (25.8)	282 (74.2)		81 (21.4%)	298 (78.6%)	
Average annual household income (RMB)	< 30,000	1.91 ± 0.44	0.241	14 (23.7)	45 (76.3)	0.669	10 (16.9%)	49 (83.1%)	0.574
	30,000–70,000	1.95 ± 0.41		51 (25.8)	147 (74.2)		46 (23.2%)	152 (76.8%)	
	>70,000	2.00 ± 0.42		54 (28.9)	133 (71.1)		39 (21.0%)	147 (79.0%)	
Father's education level	Primary school or below	1.85 ± 0.48^ab^	0.010^*^	5 (19.2) ^ab^	21 (80.8) ^ab^	0.003^**^	6 (22.2%)	21 (77.8%)	0.222
	Middle School	1.93 ± 0.39^a^		38 (18.6)^b^	166 (81.4)^b^		34 (16.7%)	170 (83.3%)	
	High school	2.06 ± 0.45^b^		48 (32.7)^a^	99 (67.3)^a^		37 (25.3%)	109 (74.7%)	
	College or above	1.93 ± 0.38^ab^		28 (36.8)^a^	48 (63.2)^a^		18 (24.0%)	57 (76.0%)	
Mother's education level	Primary school or below	1.86 ± 0.38^a^	0.016^*^	9 (13.0)^a^	60 (87.0)^a^	< 0.001^***^	8 (11.4%)	62 (88.6%)	0.080
	Middle School	1.93 ± 0.39^ab^		30 (19.0)^ab^	128 (81.0)^ab^		33 (21.0%)	124 (79.0%)	
	High school	2.02 ± 0.43^b^		43 (30.9) ^bc^	96 (69.1) ^bc^		30 (21.7%)	108 (78.3%)	
	College or above	2.03 ± 0.45^ab^		38 (43.2)^c^	50 (56.8)^c^		25 (28.4%)	63 (71.6%)	
Classification of BMI	Underweight	1.90 ± 0.34	0.627	5 (17.2)	24 (82.8)	0.424	6 (20.7%)	23 (79.3%)	0.470
	Normal weight	1.98 ± 0.44		90 (28.5)	226 (71.5)		60 (19.1%)	254 (80.9%)	
	Overweight	1.94 ± 0.39		15 (24.6)	46 (75.4)		14 (23.0%)	47 (77.0%)	
	Obesity	1.92 ± 0.38		11 (21.2)	41 (78.8)		15 (28.3%)	38 (71.7%)	
**Characteristics**	**Group**	**Fried food intake** ^3^		**Dessert intake** ^3^		**Sugary beverage intake** ^3^	
		**Median (IQR)**	***p***	**Median (IQR)**	***p***	**Median (IQR)**	***p***
Gender	Boys	9.9 (14.7)	0.338	6.7 (18.0)	0.006^**^	42.8 (101.9)	< 0.001^***^
	Girls	6.7 (14.8)		13.4 (36.1)		23.5 (29.4)	
Age	Thirteen	6.7 (18.0)	0.365	10.7 (39.4)	0.402	23.5 (61.5)	0.120
	Fourteen	10.7 (14.7)		10.7 (21.6)		26.8 (61.5)	
	Fifteen	6.7 (13.7)		10.7 (43.3)		36.9 (95.5)	
Household registration	Urban	6.7 (18.0)	0.843	12.1 (44.8)	0.774	23.5 (61.5)	0.365
	Rural	6.7 (14.7)		10.7 (36.8)		36.9 (61.5)	
Average annual household income (RMB)	< 30,000	6.7 (10.0)^a^	0.029^*^	8.7 (32.3)^a^	0.043^*^	13.4 (29.9) ^a^	0.001^**^
	30,000–70,000	6.7 (13.5)^ab^		10.7 (21.6)^a^		36.9 (61.5)^b^	
	>70,000	13.2 (14.7)^b^		10.7 (43.3)^a^		36.9 (86.6)^b^	
Father's education level	Primary school or below	6.7 (17.6)	0.691	6.7 (18.0)	0.275	36.9 (86.6)	0.867
	Middle School	6.7 (14.7)		10.7 (21.6)		26.8 (61.5)	
	High school	6.7 (18.0)		10.7 (39.4)		23.5 (72.2)	
	College or above	10.7 (14.7)		10.7 (43.3)		42.8 (72.2)	
Mother's education level	Primary school or below	6.7 (11.8)	0.609	10.7 (17.2)	0.110	23.5 (62.0)	0.050
	Middle School	6.7 (14.8)		10.7 (21.6)		36.9 (86.6)	
	High school	10.3 (18.0)		10.7 (43.3)		36.9 (61.5)	
	College or above	6.7 (11.7)		13.4 (43.3)		23.5 (61.5)	
Classification of BMI	Underweight	13.4 (18.0)	0.325	10.7 (43.3)	0.317	36.9 (61.5)	0.369
	Normal weight	6.7 (14.7)		10.7 (32.6)		23.5 (61.5)	
	Overweight	13.4 (18.0)		10.7 (39.4)		42.8 (99.9)	
	Obesity	6.7 (6.7)		6.7 (39.5)		42.8 (61.5)	

^*^p<*0.05*, ^**^p<*0.01*, ^***^p < 0.001. ^abc^Common superscripts indicated no significant differences between the groups (*p* > 0.05). ^1^The score of physical activity was normally distributed, so t-test or one-way ANOVA was performed. ^2^Chi-square test was performed. ^3^The intake of fried food, dessert, and sugary beverages was non-normal distribution, so Mann-Whitney U-test or Kruskal-Wallis H-test was performed.

In the aspect of fruit intake, there were significant age differences and parents' education level differences in fruit intake. Thirteen-year-old participants were more likely to meet the recommended fruit intake (*p* = 0.001). The participants whose fathers had high school education level or above were more probable to meet the recommended fruit intake than those whose fathers' education level was middle school (*p* = 0.003). Compared with the participants whose mothers had high school education level or above, those whose mothers' education level was primary school or below were more challenging to meet the recommended fruit intake (*p* < 0.001). However, the differences in vegetable intake were not statistically significant across all demographic characteristics.

Regarding the fried food intake, participants with an average annual household income of > 70,000 RMB had higher intake of fried food compared to those with an average household income of < 30,000 RMB (*p* = 0.029). Likewise, there was a statistically significant difference in dessert intake based on the average annual household income (*p* = 0.043). However, there was no significant difference between the groups. Moreover, participants with an average annual household income of < 30,000 RMB had lower intake of sugary beverages than those with an average household income of > 30,000 RMB (*p* = 0.001). Compared to boys, girls were more likely to consume more dessert and fewer sugary beverages (*p* = 0.006 and *p* < 0.001, respectively).

### 3.3 Associations between physical activity, dietary behaviors, and body appreciation

Correlation analysis (Table 3) showed that body appreciation was positively correlated with physical activity (*r* = 0.346, *p* < 0.01), sufficient fruit intake (*r* = 0.142, *p* < 0.01), and sufficient vegetable intake (*r* = 0.101, *p* < 0.05). Conversely, a significant negative correlation was observed between body appreciation and the intake of fried food (*r* = −0.112, *p* < 0.05).

**Table 3:** Correlation between body appreciation, physical activity, and dietary behaviors.

	**Body appreciation**	**Physical activity**	**Fruit intake**	**Vegetable intake**	**Fried food intake**	**Dessert intake**	**Sugary beverage intake**
Body appreciation	1						
Physical activity	0.346^**^	1					
Fruit intake	0.142^**^	0.236^*^	1				
Vegetable intake	0.101^*^	0.163^*^	0.336^**^	1			
Fried food intake	−0.112^*^	−0.086	0.091	−0.048	1		
Dessert intake	0.047	−0.002	0.169^**^	0.001	0.420^**^	1	
Sugary beverage intake	0.010	0.035	−0.027	−0.049	0.475^**^	0.367^**^	1

^*^p < 0.05, ^**^p < 0.01. Pearson correlation was performed to analyze the correlation between body appreciation and physical activity; Spearman correlation was performed to analyze the correlation between body appreciation and the intake of fruit, vegetables, fried food, dessert, and sugary beverages.

According to the results of univariate analysis, gender, age, parents' education level, average annual household income, and body appreciation were included in the logistic regression model of dietary behaviors (Table 4). There was a statistically significant trend for adequate fruit and vegetable consumption among adolescents with more educated mothers compared to those where mothers only had primary school or below education (both *p* < 0.05). Participants with higher body appreciation were more likely to meet the recommended fruit and vegetable intake [aO*R* = 1.45, 95%CI (1.10–1.90), *p* < 0.01; aO*R* = 1.34, 95%CI (1.01–1.76), *p* < 0.05, respectively].

**Table 4:** Associations between dietary behaviors, physical activity, and body appreciation.

	**Fruit intake**		**Vegetable intake**		**Fried food intake** ^**1**^		**Physical activity**	
	**Model 1 OR (95%CI)**	**Model 2 aOR (95%CI)**	**Model 1 OR (95%CI)**	**Model 2 aOR (95%CI)**	**Model 1** β **(95%CI)**	**Model 2** β **(95%CI)**	**Model 1** β **(95%CI)**	**Model 2** β **(95%CI)**
Body appreciation	1.43 (1.11 to 1.85)^**^	1.45 (1.10 to 1.90)^**^	1.36 (1.03 to 1.78)^*^	1.34 (1.01 to 1.76)^*^		−0.08 (−0.13 to −0.03)^**^		0.16 (0.12 to 0.20)^***^
Age		0.57 (0.41 to 0.80)^**^		1.11 (0.79 to 1.56)	0.03 (−0.03 to 0.09)	0.03 (−0.03 to 0.09)		
**Gender**								
Boys		Ref.		Ref.	Ref.	Ref.	Ref.	Ref.
Girls		1.09 (0.69 to 1.71)		0.92 (0.58 to 1.48)	−0.05 (−0.14 to 0.04)	−0.06 (−0.14 to 0.03)	−0.14 (−0.21 to −0.06)^***^	−0.12 (−0.20 to −0.05)^**^
**Father's education level**								
Primary school or below		Ref.		Ref.	Ref.	Ref.	Ref.	Ref.
Middle School		0.57 (0.18 to 1.80)		0.43 (0.13 to 1.37)	0.15 (−0.05 to 0.36)	0.17 (−0.04 to 0.37)	0.04 (−0.13 to 0.22)	0.00 (−0.16 to 0.17)
High school		0.96 (0.29 to 3.24)		0.73 (0.22 to 2.49)	0.07 (−0.15 to 0.29)	0.08 (−0.14 to 0.30)	0.12 (−0.07 to 0.31)	0.10 (−0.08 to 0.28)
College or above		0.81 (0.22 to 3.01)		0.56 (0.15 to 2.12)	0.11 (−0.14 to 0.36)	0.12 (−0.13 to 0.36)	−0.04 (−0.25 to 0.17)	−0.07 (−0.26 to 0.13)
**Mother's education level**								
Primary school or below		Ref.		Ref.	Ref.	Ref.	Ref.	Ref.
Middle School		1.73 (0.69 to 4.29)		3.13 (1.15 to 8.54)^*^	0.05 (−0.10 to 0.19)	0.07 (−0.07 to 0.21)	0.06 (−0.06 to 0.19)	0.02 (−0.09 to 0.14)
High school		2.99 (1.16 to 7.71)^*^		2.85 (0.99 to 8.24)	0.09 (−0.07 to 0.24)	0.11 (−0.05 to 0.26)	0.13 (−0.01 to 0.26)	0.09 (−0.04 to 0.22)
College or above		4.15 (1.49 to 11.60)^**^		3.75 (1.19 to 11.80)^*^	0.05 (−0.13 to 0.23)	0.08 (−0.10 to 0.26)	0.19 (0.03 to 0.34)^*^	0.13 (−0.02 to 0.28)
**Average annual household income (RMB)**								
< 30000		Ref.		Ref.	Ref.	Ref.		
30,000–70,000		0.95 (0.45 to 1.97)		1.24 (0.57 to 2.73)	0.08 (−0.06 to 0.22)	0.07 (−0.07 to 0.21)		
>70,000		0.90 (0.42 to 1.90)		0.93 (0.41 to 2.10)	0.16 (0.01 to 0.30)^*^	0.17 (0.02 to 0.31)^*^		
*R* ^2^					0.033	0.055	0.066	0.174
Δ*R*^2^					0.033	0.021	0.066	0.108
*F*					1.480	2.240^*^	4.529^***^	11.794^***^

^*^p < 0.05, ^**^p < 0.01, ^***^p < 0.001. 95% CI: 95% confidence interval; Ref: reference group. ^1^A natural logarithm conversion was carried out in order to make the fried food intake become approximately normally distributed data.

Table 4 also showed that participants with an average annual household income of >70,000 RMB had significantly higher fried food intake than those with an annual household income of < 30,000 RMB [β = 0.17, 95%CI (0.02–0.31), *p* < 0.05]. Meanwhile, hierarchical linear analysis of fried food intake indicated that body appreciation explained an additional 2.1% of the variance in participants' fried food intake when controlling for demographic covariates (*R*^2^ = 0.055, *F* = 2.240, *p* < 0.05). More specifically, participants with higher body appreciation were less likely to consume fried food [β = −0.08, 95%CI (−0.13 to −0.03), *p* < 0.01].

The association between body appreciation and physical activity was also explored using hierarchical linear regressions. As shown in Table 4, demographic characteristics, including gender and parents' education level, were entered into Model 1, accounting for a variance of 6.6% (*R*^2^ = 0.066, *F* = 4.529, *p* < 0.001). In Model 2, body appreciation explained an additional 10.8% of the variance in physical activity when controlling for demographic characteristics (*R*^2^ = 0.174, *F* = 11.794, *p* < 0.001). In summary, girls [β = −0.12, 95%CI (−0.20 to −0.05), *p* < 0.01] were less likely to engage in physical activity and those with higher body appreciation [β = 0.16, 95%CI (0.12–0.20), *p* < 0.001] were more likely to engage in physical activity.

## 4 Discussion

This study investigated the current status of body appreciation and weight-related health behaviors among Chinese adolescents and contributed novel information about this group. The body appreciation measured by the BAS-2 in this study was lower than Canadian adolescents (Paquette et al., 2022), but higher than Lithuanian adolescents (Baceviciene and Jankauskiene, 2020). According to sociocultural theory (Cash, 2005), cultural attitudes toward body image and societal pressures unique in each country may explain the differences in body appreciation among adolescents from various cultural backgrounds. Within China, regional variations in body appreciation were also observed. Compared with adolescents in Wuhan (Chen et al., 2020) and Dandong (Gao et al., 2022), the adolescents in Hangzhou had a lower score of body appreciation. These differences may be influenced by regional disparities in lifestyle, socioeconomic status, and access to health education. Adolescents in urban areas of economically developed cities like Hangzhou may experience higher societal pressures related to body image due to greater exposure to media and social “ideal figure” expectations (Duan et al., 2022). Meanwhile, our participant adolescents' physical activity was lower than adolescents in Guilin (Lu et al., 2022) and Ethiopia (Andarge et al., 2021). Particularly, most of them were categorized as inactive, which indicated a poor level of physical activity. Likewise, unhealthy dietary behaviors were discovered in participants, such as insufficient intake of fruit and vegetables or excessive intake of fried food, dessert, and sugary beverages. Both insufficient physical activity and unhealthy eating habits among adolescents contribute to excessive weight gain, resulting in irreversible physiological and psychological harm to this age group.

Numerous studies have reported a positive correlation between body appreciation and physical activity (August et al., 2023; Shi et al., 2024; Zhou et al., 2024). Our findings also suggested that body appreciation was positively associated with physical activity in Chinese adolescents, which was consistent with Wang's study (Wang, 2022). Self-determination theory (Ryan and Deci, 2000) (SDT) could provide a theoretical mechanism for interpreting how body appreciation may influence physical activity, with its focus on the motivation underlying human behavior (Hurst et al., 2017). Specifically, Cox et al. (2019) linked body image variables to the intrinsic regulation of physical activity as defined within SDT, and discovered that body appreciation was a significant predictor of intrinsic motivation to be physically active. Therefore, individuals who maintain body appreciation are more likely to generate motivation to participate in physical activity.

Domestic scholars primarily investigated the relationship between body appreciation and intuitive eating (Luo et al., 2019) or eating disorders (Luo et al., 2021) among young women or adults, with little research exploring the relationship between body appreciation and daily dietary behaviors among adolescents. In this study, we found that body appreciation was positively associated with sufficient fruit and vegetable intake, while negatively associated with fried food intake. Consuming sufficient fruit and vegetables and reducing the intake of fried food epitomized healthy eating. In other words, body appreciation was associated with healthy dietary behaviors among adolescents, which has been confirmed in foreign studies (Jalali-Farahani et al., 2022; Neumark-Sztainer et al., 2006) as well. Individuals with low body appreciation may suffer from feelings of shame or self-criticism, based upon Heatherton and Baumeister's escape theory (Heatherton and Baumeister, 1991), they will turn to unhealthy eating behaviors, such as consuming high-fat, high-calorie foods to cope with those negative emotions, shifting from threatening stimuli to pleasurable ones. Consequently, it suggests that we can motivate adolescents to adopt healthy dietary behaviors by reinforcing the construction of body appreciation.

Apart from body appreciation, physical activity and dietary behaviors varied by adolescent sex and family socio-economic status (SES). In detail, girls participated in physical activity less frequently than boys, which was in keeping with those from the majority of similar surveys (Cairney et al., 2012). Also, maternal education level was found have a more prominent impact than body appreciation on teenagers adopting healthy dietary behaviors (including sufficient fruit and vegetable consumption). Compared to body appreciation, higher annual household income significantly affected the consumption of fried food among adolescents, likely because those from high-income families have greater access to fried food without financial constraints. Therefore, parent education is also important targets of interventions aimed at improving dietary behaviors of adolescents.

Physical activity and dietary behaviors are significant factors influencing weight management in adolescents. Therefore, this study focused on the relationship between body appreciation and weight-related health behaviors (including physical activity and dietary behaviors), a topic hitherto unexplored among Chinese adolescents. Our study revealed that adolescents with high body appreciation were more likely to engage in healthy eating and physical activity, developing and maintaining healthy lifestyles and weight management throughout the life cycle. Therefore, body appreciation during adolescence should be given special attention. Initially, adolescents should cultivate body appreciation and proper weight perceptions through targeted interventions, such as thematic educational programs. Then, the family and school environments wield substantial influence over adolescents' character development and cognitive formation (Sugiarti et al., 2022; Verhoeven et al., 2019). Parents should encourage open and honest communication about body image and emphasize the uniqueness and individuality of each person's body, helping adolescents develop correct and healthy aesthetics. Simultaneously, schools should expand their curriculum to include more courses and lectures on body image, nurturing adolescents' respectful outlook toward their own bodies and those of their peers. This multifaceted approach enhances their comprehension of body image dynamics and lays a robust foundation for cultivating genuine body appreciation.

There are several strengths and limitations in this study. This study provided novel information on the current status of body appreciation, physical activity, and dietary behaviors among Chinese adolescents. The study also explored the relationships between body appreciation and weight-related health behaviors in Chinese adolescents, which has received little attention in previous research. Some limitations still need to be addressed. Firstly, the cross-sectional design hinders conclusive inferences about causality, therefore, longitudinal and qualitative research could be conducted in the future (Wang and Cheng, 2020). Secondly, our findings are only confirmed for adolescents aged 13–15 living in Hangzhou, China, and thus generalizability is limited. Thirdly, for the convenience of the questionnaire, only fruit, vegetables, fried food, dessert, and sugary beverages were selected as indicators of adolescents' dietary behaviors. Twenty-four hours dietary recall method can be employed in further studies to investigate dietary consumption thoroughly among adolescents (Salvador Castell et al., 2015).

## 5 Conclusions

The study shows current status of body appreciation among Chinese adolescents, with a majority categorized as physically inactive and having unhealthy dietary behaviors, leading to abnormal weight. Notably, this study provides novel evidence linking body appreciation with positive weight-related behavioral outcomes in both physical activity and dietary behaviors (such as sufficient fruit and vegetable consumption and reduced fried food intake) among Chinese adolescents. Therefore, the impact of body appreciation should be taken into account when devising strategies to improve weight-related health behaviors in this population. Besides, this study also reveals the impact of gender, mother's education, and household income on weight-related health behaviors among adolescents. These findings present empirical evidence for the development of intervention programs aimed at fostering body appreciation and promoting weight-related health behaviors in adolescents.

## Data Availability

The raw data supporting the conclusions of this article will be made available by the authors, without undue reservation.

## References

[B1] Abarca-Gómez L. Abdeen Z. Hamid Z. Abu-Rmeileh N. Acosta-Cazares B. Acuin C (2017). Worldwide trends in body-mass index, underweight, overweight, and obesity from 1975 to 2016: a pooled analysis of 2416 population-based measurement studies in 128·9 million children, adolescents, and adults. Lancet 390, 2627–2642. 10.1016/S0140-6736(17)32129-3 29029897 PMC5735219

[B2] Andarge E. Trevethan R. Fikadu T (2021). Assessing the physical activity questionnaire for adolescents (PAQ-A): specific and general insights from an Ethiopian context. Biomed Res. Int. 2021:5511728. 10.1155/2021/5511728 34337016 PMC8294967

[B3] August K. J. Malik D. Markey C. H. Woods K. Gerwitz G. C. (2023). Additive and interactive associations among body appreciation, self-compassion, and gender in understanding college students' health behaviors. Body Image 47:101634. 10.1016/j.bodyim.2023.101634 37774424

[B4] Baceviciene M. Jankauskiene R. (2020). Associations between body appreciation and disordered eating in a large sample of adolescents. Nutrients 12:752. 10.3390/nu12030752 32178334 PMC7146197

[B5] Benítez-Porres J. Alvero-Cruz J. R. Sardinha L. B. López-Fernández I. Carnero E. A. (2016). Cut-off values for classifying active children and adolescentes using the physical activity questionnaire: PAQ-C and PAQ-ACut-off values for classifying active children and adolescents using the physical activity questionnaire: PAQ-C and PAQ-A. Nutricion Hospitalaria 33:564. 10.20960/nh.564 27759968

[B6] Cairney J. Kwan M. Y. Velduizen S. Hay J. Bray S. R. Faught B. E. (2012). Gender, perceived competence and the enjoyment of physical education in children: a longitudinal examination. Int. J. Beha. Nutr. Phys. Act. 9:26. 10.1186/1479-5868-9-26 22394618 PMC3310805

[B7] Carvalho G. X. Nunes A. P. N. Moraes C. L. Veiga G. V. D. (2020). Body image dissatisfaction and associated factors in adolescents. Ciencia Saude Coletiva 25, 2769–2782. 10.1590/1413-81232020257.27452018 32667558

[B8] Cash T. F. (2005). The influence of sociocultural factors on body image: searching for constructs. Clin. Psychol. Sci. Pract. 12, 438–442. 10.1093/clipsy.bpi05529535079

[B9] Cash T. F. Fleming E. C. (2002). The impact of body image experiences: development of the body image quality of life inventory. Int. J. Eat. Disord. 31, 455–460. 10.1002/eat.10033 11948650

[B10] Cash T. F. Pruzinsky T. Grazer F. M. Sorensen C. L. (1991). Body images: development, deviance, and change. Plastic Reconstruct. Surg. 88, 367. 10.1097/00006534-199108000-00041

[B11] Chen X. Luo Y. Chen H. (2020). Friendship quality and adolescents' intuitive eating: a serial mediation model and the gender difference. Acta Psychologica Sinica 52, 485–496. 10.3724/SP.J.1041.2020.0048537113526

[B12] Cheng Z. Shuai P. Qiao Q. Li T. (2020). Validity and reliability of a simplified food frequency questionnaire: a cross sectional study among physical health examination adults in southwest region of China. Nutr. J. 19:114. 10.1186/s12937-020-00630-z 33023588 PMC7541293

[B13] Chinese Nutrition Society. (2022). Dietary Guidelines for Chinese School-Aged Children (2022). Beijing: People's Medical Publishing House.

[B14] Cox A. E. Ullrich-French S. Tylka T. L. McMahon A. K. (2019). The roles of self-compassion, body surveillance, and body appreciation in predicting intrinsic motivation for physical activity: cross-sectional associations, and prospective changes within a yoga context. Body Image 29, 110–117. 10.1016/j.bodyim.2019.03.002 30921763

[B15] Daly A. N. O'Sullivan E. J. Kearney J. M. (2022). Considerations for health and food choice in adolescents. Proc. Nutr. Soc. 81, 75–86. 10.1017/S0029665121003827 35039094

[B16] Duan C. Lian S. Yu L. Niu G. Sun X. (2022). Photo activity on social networking sites and body dissatisfaction: the roles of thin-ideal internalization and body appreciation. Behav. Sci. 12:280. 10.3390/bs12080280 36004851 PMC9404895

[B17] Gao Y. Liu S. Yu X. Huang W. Zhao L. Teng J.. (2022). Sociocultural attitudes towards appearance on unhealthy weight controlbehaviors: the mediating effects of body appreciation and lntuitive eating. Chin. J. Clin. Psychol. 30, 554–560. 10.16128/j.cnki.1005-3611.2022.03.012

[B18] Gattario K. H. Frisén A. (2019). From negative to positive body image: Men's and women's journeys from early adolescence to emerging adulthood. Body Image 28, 53–65. 10.1016/j.bodyim.2018.12.002 30583277

[B19] Gualdi-Russo E. Rinaldo N. Zaccagni L. (2022). Physical activity and body image perception in adolescents: a systematic review. Int. J. Environ. Res. Public Health 19:13190. 10.3390/ijerph192013190 36293770 PMC9603811

[B20] He J. Cui T. Barnhart W. R. Chen G. (2023). The Chinese version of the functionality appreciation scale: psychometric properties and measurement invariance across gender and age. J. Eat. Disord. 11:99. 10.1186/s40337-023-00826-8 37340301 PMC10283255

[B21] Heatherton T. F. Baumeister R. F. (1991). Binge eating as escape from self-awareness. Psychol. Bull. 110, 86–108. 10.1037/0033-2909.110.1.86 1891520

[B22] Hong J. Gong Q. Gao H. Wang J. Guo Y. Jiang D.. (2023). Association between dietary behavior and overweight and obesity among Chinese students: a cross-sectional study. Children 10:1617. 10.3390/children10101617 37892280 PMC10605267

[B23] Hurst M. Dittmar H. Banerjee R. Bond R. (2017). “I just feel so guilty”: the role of introjected regulation in linking appearance goals for exercise with women's body image. Body Image 20, 120–129. 10.1016/j.bodyim.2016.12.002 28193554

[B24] Jalali-Farahani S. Zayeri F. Zarani F. Azizi F. Amiri P. (2022). Network associations among body image, lifestyle, body mass index, and quality of life in adolescents. Int. J. Endocrinol. Metabol. 20:e123237. 10.5812/ijem-123237 35993035 PMC9375937

[B25] Jiménez Flores P. Jiménez Cruz A. Bacardi Gascón M. (2017). Body-image dissatisfaction in children and adolescents: a systematic review. Nutricion Hospitalaria 34, 479–489. 10.20960/nh.455 28421808

[B26] Kantanista A. Osiński W. Borowiec J. Tomczak M. Król-Zielińska M. (2015). Body image, BMI, and physical activity in girls and boys aged 14-16 years. Body Image 15, 40–43. 10.1016/j.bodyim.2015.05.001 26047066

[B27] Lei Y. T. Ma J. Hu P. J. Dong B. Zhang B. Song Y. (2019). [The status of spermarche, menarche and corresponding relationships with nutritional status among students of 13 ethnic minorities in Southwest China in 2014]. Chin. J. Prevent. Med. 53, 492–496. 10.3760/cma.j.issn.0253-9624.2019.05.011 31091607

[B28] Li L. Ouyang Y. Wang H. Huang F. Wang Y. Zhang J.. (2020). Status of fruit and vegetable intake among children and adolescents in 15 provinces of China. Chin. J. Health Educ. 36, 3–7.

[B29] Li X. Wang Y. Li X. Li D. Sun C. Xie M.. (2015). Reliability and Validity of Physical Activity Questionnaire for Adolescents(PAQ-A) in Chinese Version. J. Beijing Sport Univ. 38, 63–67.39529046

[B30] Li Z. Wu H. Liangwu Q. (2019). Assessment of physical activity level, health status and perceptual impairment in adolescent students. J. Kunming Med. Univ. 40, 39–43.

[B31] Linardon J. (2021). Positive body image, intuitive eating, and self-compassion protect against the onset of the core symptoms of eating disorders: a prospective study. Int. J. Eat. Disord. 54, 1967–1977. 10.1002/eat.23623 34599619

[B32] Linardon J. McClure Z. Tylka T. L. Fuller-Tyszkiewicz M. (2022). Body appreciation and its psychological correlates: A systematic review and meta-analysis. Body Image 42, 287–296. 10.1016/j.bodyim.2022.07.003 35878528

[B33] Lu H. Wang H. Chen Q. Wei H. (2022). Analysis of the current situation and influencing factors of physical activity among secondary school students in Guilin. Sci. Technol. Station. Sport. Goods 24, 127–129.

[B34] Luo Y. J. Jackson T. Stice E. Chen H. (2021). Effectiveness of an internet dissonance-based eating disorder prevention intervention among body-dissatisfied young Chinese women. Behav. Ther. 52, 221–233. 10.1016/j.beth.2020.04.007 33483119

[B35] Luo Y. J. Niu G. F. Kong F. C. Chen H. (2019). Online interpersonal sexual objectification experiences and Chinese adolescent girls' intuitive eating: the role of broad conceptualization of beauty and body appreciation. Eat. Behav. 33, 55–60. 10.1016/j.eatbeh.2019.03.004 30927695

[B36] McClain Z. Peebles R. (2016). Body image and eating disorders among lesbian, gay, bisexual, and transgender youth. Pediatr. Clin. North Am. 63, 1079–1090. 10.1016/j.pcl.2016.07.008 27865334 PMC6402566

[B37] Neumark-Sztainer D. Paxton S. J. Hannan P. J. Haines J. Story M. (2006). Does body satisfaction matter? Five-year longitudinal associations between body satisfaction and health behaviors in adolescent females and males. J. Adolesc. Health 39, 244–251. 10.1016/j.jadohealth.2005.12.001 16857537

[B38] Nolen E. Panisch L. S. (2022). The relationship between body appreciation and health behaviors among women and adolescent girls: a scoping review. Health Soc. Work 47, 113–122. 10.1093/hsw/hlac006 35262682

[B39] Paquette M. M. Dion J. Bothe B. Bergeron S. (2022). Validation of the body appreciation scale-2 in cisgender, heterosexual and sexual and gender minority adolescents and sexuality-related correlates. Body Image 43, 193–204. 10.1016/j.bodyim.2022.09.001 36154978

[B40] Piko B. F. Obál A. Mellor D. (2020). Body appreciation in light of psychological, health- and weight-related variables among female adolescents. Europe's J. Psychol. 16, 676–687. 10.5964/ejop.v16i4.2183 33680205 PMC7909497

[B41] Riddervold S. Haug E. Kristensen S. M. (2023). Sports participation, body appreciation and life satisfaction in Norwegian adolescents: a moderated mediation analysis. Scand. J. Public Health 52, 704–710. 10.1177/14034948231184525 37403364

[B42] Ryan R. M. Deci E. L. (2000). Self-determination theory and the facilitation of intrinsic motivation, social development, and well-being. Am. Psychol. 55, 68–78. 10.1037/0003-066X.55.1.68 11392867

[B43] Salvador Castell G. Serra-Majem L. Ribas-Barba L. (2015). What and how much do we eat? 24-hour dietary recall method. Nutricion Hospitalaria 31, 46–48. 10.3305/nh.2015.31.sup3.8750 25719770

[B44] Shi L. Jiang L. Zhou S. Zhou W. Yang H. (2024). Self-appreciation is not enough: exercise identity mediates body appreciation and physical activity and the role of perceived stress. Front. Psychol. 15:1377772. 10.3389/fpsyg.2024.1377772 39319073 PMC11420792

[B45] Song L. Zhang Y. Chen T. Maitusong P. Lian X. (2022). Association of body perception and dietary weight management behaviours among children and adolescents aged 6–17 years in China: cross-sectional study using CHNS (2015). BMC Public Health 22:175. 10.1186/s12889-022-12574-6 35081917 PMC8790848

[B46] Sugiarti R. Erlangga E. Suhariadi F. Winta M. V. I. Pribadi A. S. (2022). The influence of parenting on building character in adolescents. Heliyon 8:e09349. 10.1016/j.heliyon.2022.e09349 35586332 PMC9108886

[B47] Swami V. Ng S. K. Barron D. (2016). Translation and psychometric evaluation of a Standard Chinese version of the body appreciation scale-2. Body Image 18, 23–26. 10.1016/j.bodyim.2016.04.005 27236474

[B48] Tylka T. L. Wood-Barcalow N. L. (2015a). The body appreciation scale-2: item refinement and psychometric evaluation. Body Image 12, 53–67. 10.1016/j.bodyim.2014.09.006 25462882

[B49] Tylka T. L. Wood-Barcalow N. L. (2015b). What is and what is not positive body image? Conceptual foundations and construct definition. Body Image 14, 118–129. 10.1016/j.bodyim.2015.04.001 25921657

[B50] Verhoeven M. Poorthuis A. Volman M. (2019). The role of school in adolescents' identity development. a literature review. Educ. Psychol. Rev. 31, 35–63. 10.1007/s10648-018-9457-3

[B51] Wang X. Cheng Z. (2020). Cross-sectional studies: strengths, weaknesses, and recommendations. Chest 158, S65–S71. 10.1016/j.chest.2020.03.012 32658654

[B52] Wang F. Li Y. Feng Q. (2021). Status quo and characteristics of physical activities of children and adolescents. Afterschool Educ. China 22, 86–107.

[B53] Wang G. (2022). Influence of body image on physical exercise of adolescents of different genders. Shandong Sports Sci. Technol. 44, 51–57. 10.14105/j.cnki.1009-9840.2022.03.004

[B54] Wyszyńska J. Matłosz P. Podgórska-Bednarz J. Herbert J. Przednowek K. Baran J.. (2019). Adaptation and validation of the physical activity questionnaire for adolescents (PAQ-A) among Polish adolescents: cross-sectional study. BMJ Open 9:e030567. 10.1136/bmjopen-2019-030567 31740466 PMC6887051

[B55] Zhou S. Guan Q. Feng K. Leng M. Ma X. Zhou W. (2024). Longitudinal relationships between physical activity, body appreciation, and proactive coping in college students: a cross-lagged panel model. Body Image 51:101814. 10.1016/j.bodyim.2024.101814 39531754

